# Tracking the NGS revolution: managing life science research on shared high-performance computing clusters

**DOI:** 10.1093/gigascience/giy028

**Published:** 2018-04-05

**Authors:** Martin Dahlö, Douglas G Scofield, Wesley Schaal, Ola Spjuth

**Affiliations:** 1 Science for Life Laboratory, Uppsala University, Uppsala, SE-750 03, Sweden; 2 Uppsala Multidisciplinary Center for Advanced Computational Science, Uppsala University, Uppsala, SE-751 05, Sweden; 3 Department of Pharmaceutical Biosciences, Uppsala University, Uppsala, SE-751 24, Sweden; 4 Department of Ecology and Genetics: Evolutionary Biology, Uppsala University, Uppsala, SE-752 36, Sweden

**Keywords:** high-performance computing, e-infrastructures, bioinformatics, resource usage efficiency, efficiency metrics, storage, next generation sequencing

## Abstract

**Background:**

Next-generation sequencing (NGS) has transformed the life sciences, and many research groups are newly dependent upon computer clusters to store and analyze large datasets. This creates challenges for e-infrastructures accustomed to hosting computationally mature research in other sciences. Using data gathered from our own clusters at UPPMAX computing center at Uppsala University, Sweden, where core hour usage of ∼800 NGS and ∼200 non-NGS projects is now similar, we compare and contrast the growth, administrative burden, and cluster usage of NGS projects with projects from other sciences.

**Results:**

The number of NGS projects has grown rapidly since 2010, with growth driven by entry of new research groups. Storage used by NGS projects has grown more rapidly since 2013 and is now limited by disk capacity. NGS users submit nearly twice as many support tickets per user, and 11 more tools are installed each month for NGS projects than for non-NGS projects. We developed usage and efficiency metrics and show that computing jobs for NGS projects use more RAM than non-NGS projects, are more variable in core usage, and rarely span multiple nodes. NGS jobs use booked resources less efficiently for a variety of reasons. Active monitoring can improve this somewhat.

**Conclusions:**

Hosting NGS projects imposes a large administrative burden at UPPMAX due to large numbers of inexperienced users and diverse and rapidly evolving research areas. We provide a set of recommendations for e-infrastructures that host NGS research projects. We provide anonymized versions of our storage, job, and efficiency databases.

## Background

Ever since the development of next-generation sequencing (NGS) technology, biology has become increasingly data-intensive [1]. The number of research groups working with large amounts of data and requiring significant computing and storage resources has grown immensely, as have the diversity of research questions for which sequence data are being used [2-5] and the sophistication of sequence data that are being generated [6,7]. Hardware resource requirements often greatly exceed those available in desktop computers. For many research groups, it is not feasible to purchase and maintain dedicated computing clusters. As a result, biologists are making increasing use of high-performance computing (HPC) centers with large amounts of computing power and storage that are shared with other users. In parallel, research groups have been developing software tools and databases to assist in the analysis of these data. Indeed, NGS method development represents a very active area of research [8,9].

To effectively conduct research, many life science researchers now need to become comfortable with command-line interaction with Linux operating systems and research-oriented software tools, which is a major change in expectations compared to just a few years ago. This contrasts strongly with expectations in research fields that have a longer history of HPC usage, such as physics, computational chemistry, and climate science research, in which the general computational sophistication of researchers and the maturity of software tools are both considerably higher [e.g., 10].

In Sweden, six large academic HPC centers are managed by the Swedish National Infrastructure for Computing (SNIC), which is responsible for planning, funding, and organizing academic HPC resources. These resources are provided at no cost to research groups in Swedish academia. The SNIC center, located at Uppsala University, is the Uppsala Multidisciplinary Center for Advanced Computational Science, or UPPMAX, at which are found several computing clusters and high-performance storage systems ( Tables 1 and 2). As with other SNIC centers, UPPMAX hosts HPC resources used for general computationally intensive academic research. As a result of targeted development, UPPMAX also hosts the HPC resources used for most NGS-related academic research in Sweden.

**Table 1:** UPPMAX high-performance computing clusters available for NGS and non-NGS projects during the study period, some providing higher-memory nodes available at user request.

HPC cluster	Nodes	Total cores	RAM/Node	Description
Kalkyl (2010-2014)	350	2,800	24 GiB; 16× 48 GiB; 16× 72 GiB	Half for NGS projects
Halvan (2011-2015)	1	64	2 TiB	NGS projects
Tintin (2012-2017)	160	2,560	64 GiB; 16× 128 GiB	Primarily non-NGS projects
Milou (2013-2017)	208	3,328	128 GiB; 17× 256 GiB; 17× 512 GiB	NGS projects
Fysast1 (2013-2017)	40	640	128 GiB	Non-NGS projects in physics and astronomy

**Table 2:** UPPMAX high-performance storage systems available for NGS and non-NGS projects during the study period

HPC storage	Capacity, PiB	Format	Description
Bubo (2009-2013)	0.7	PanFS	Shared storage
Lynx (2012-2015)	0.5	PanFS	Shared storage
Gulo (2012-2016)	1.0	Lustre	Global scratch storage
Pica (2013-2017)	5.5	NFS	Shared storage

Sweden also has a national organization responsible for facilitating the development of life science research, the Science for Life Laboratory, or SciLifeLab, located primarily in Uppsala and Stockholm. SciLifeLab contributes to funding and, in part, manages HPC systems for NGS data production and analysis, both for use by its own sequencing facilities and for use by life science researchers at Swedish universities and their international collaborators. This includes the Milou and Pica resources for research at UPPMAX that are covered here (Tables 1 and 2). SciLifeLab-managed HPC resources used for NGS data production and delivery have been discussed elsewhere [11].

Because of the novelty of the research field and the rapidly changing research and technological landscapes, an important guiding principal for management of computing resources for NGS projects at UPPMAX has been flexibility, i.e., granting temporary increases in project core hour and storage allocations, allowing long project lifetimes, and investing in user support. An active NGS project can grow considerably in computing and storage needs and have quite a long duration as new sequencing datasets are delivered, additional analyses are conducted, and new subprojects are started that depend upon derived datasets. This contrasts with management of non-NGS projects through SNIC at UPPMAX and other SNIC centers, for which monthly core hour allocations and project duration are fixed at project approval and disk storage allocations have typically been quite limited.

Here, our primary aim was to better understand the growing demands the NGS revolution has made on human and computational resources of research computing clusters. Specifically, we compare and contrast research project growth, support efforts, and cluster usage by NGS and non-NGS projects at UPPMAX. We note that for non-NGS projects, in particular, our observations at UPPMAX do not necessarily reflect usage typical at other HPC clusters in Sweden, which host non-NGS projects and jobs that are typically much larger. We describe a straightforward method for quantifying resource usage and efficiency and outline our efforts to increase the efficiency of resource usage and describe additional tools we have developed for users to use in their own evaluations. We conclude by considering the particular demands NGS projects place on UPPMAX systems and personnel and emphasize that traditional computing infrastructure is often ill prepared to handle NGS users.

## Results

Here, a *project* is a named allocation of computing and storage resources for research, with a specific principal investigator (PI) and a PI-managed set of authorized users. A project that uses NGS or NGS-derived data is an *NGS project*, while a project that does not is a *non-NGS project*. A *job* is a computing job submitted by a user via the SLURM job management system [12], which charges the core hours used by the job to a user-specified project. Jobs, users, and similar components are classified as NGS or non-NGS depending on association with an NGS or non-NGS project.

First, we compare and contrast NGS and non-NGS projects in terms of project and research group numbers, storage, support tickets, and software and resource installations. Then, we examine the profiles of computing jobs run by the different project types on UPPMAX clusters, by first comparing and contrasting resources booked for jobs via SLURM, then usage of booked cores and RAM, and finally efficiency of resource usage and our efforts to increase this efficiency.

### Rapid growth in NGS projects, research groups, and storage

**Figure 1:**
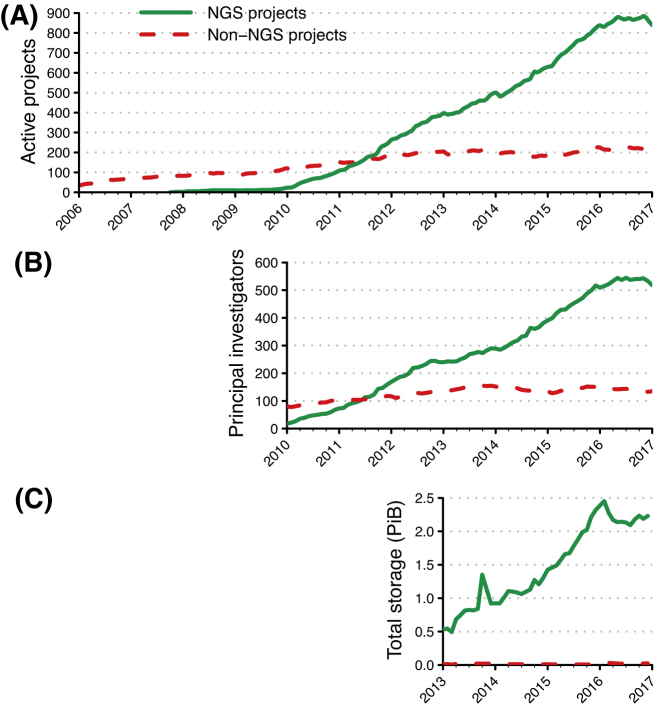
Active projects, unique project PIs, and storage used by projects on UPPMAX HPC clusters, by project type. A) Number of active NGS and non-NGS projects since 2006. B) Number of unique PIs for active NGS and non-NGS projects. C) Total amount of storage used by NGS and non-NGS projects, in pebibytes (PiB, 2^50^ bytes). The maximum storage used by non-NGS projects never exceeded 0.034 PiB in any month. The extent of the *X*-axis of each panel reflects the time period for which we have accurate data.

By the end of 2016, there were nearly four times as many active NGS projects hosted at UPPMAX as active non-NGS projects (Fig. 1A). This reflects a much higher (7.5 ×) rate of growth in active NGS projects. Since 2010, the number of NGS projects has grown by 9.8/month compared to 1.3/month for non-NGS projects.

The rapid growth in active NGS projects at UPPMAX is largely due to the entry of new research groups into NGS research, using unique PIs as a proxy for research group participation (Fig. 1B). At the end of 2016, the majority of PIs of both project types headed a single project (75.9% of NGS PIs and 87.4% of non-NGS research project PIs). For those PIs with two or more active projects, NGS PIs had more active projects than non-NGS PIs (3.55 ± 0.31 projects vs. 2.18 ± 0.10 projects, respectively; 2-sided Mann-Whitney *U* = 669.5, *P* = 0.0068; unless otherwise indicated, all means are presented as mean ± s.e.m.)

The total amount of storage used by NGS projects far exceeded that used by non-NGS projects (Fig.1C, expanding on Fig. 2B from [13]). The maximum total storage used by NGS projects during any month in 2013 through the end of 2016 was 2,511 TiB, nearly 70 × the maximum total storage of 35 TiB for all non-NGS projects. Growth in storage usage plateaued during 2016 when the storage system used for NGS projects approached its useful capacity (Fig.1C). Expansion of storage is problematic because the costs of providing high-performance storage that matches the needs of NGS projects can easily exceed the costs of the computing nodes to which the storage is attached.

**Figure 2:**
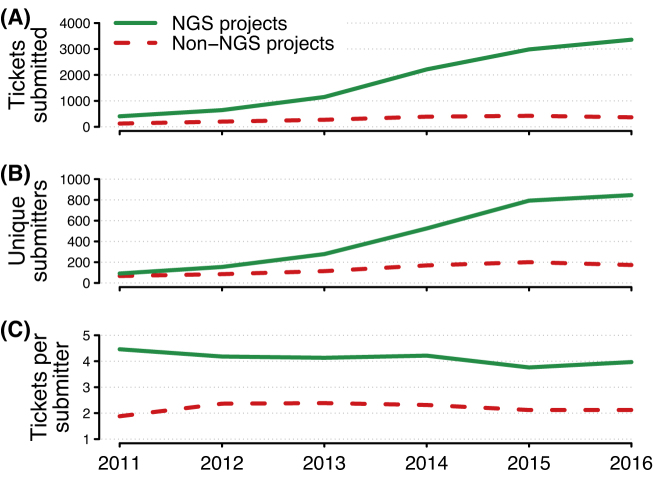
Annual support tickets submitted to UPPMAX by project type. Support tickets submitted to UPPMAX each year between 2011 and 2016, by primary project type of submitter where this can be determined. Shown are annual totals or means for each project type. A) Total number of tickets submitted. B) Number of unique ticket submitters. C) Mean number of tickets per unique submitter. See text for further methodological details.

This has necessitated more active administration of NGS project storage by UPPMAX. Since the installation of the Bubo file system in 2009 (Table 2), UPPMAX has provided NGS projects with two storage classes: with and without backup. Storage used by expired projects was reclaimed after a grace period. When PIs of active NGS projects requested renewal, storage usage was evaluated by UPPMAX. Users were encouraged to reduce their usage of backed-up storage for intermediate analyses, to remove older files that could be readily recreated or were no longer required, and to archive raw data when possible. UPPMAX also restricted maximum storage available to a single project to be 20 TiB backed-up storage and 20 TiB non-backed-up storage, with exceptions possible after further review.

### More support tickets from NGS projects and more tickets per NGS user

After filtering support tickets submitted between 2011 and 2016 using the approach described in the Methods section, we found 10,752 tickets submitted by users from NGS projects and 1,781 tickets submitted by users from non-NGS projects (Fig. 2A). For example, in 2016, NGS users submitted 3,357 tickets and non-NGS users submitted 367 tickets, a 9-fold difference. Part of the difference in ticket numbers across the years is due to the greater number of unique NGS users submitting tickets (Fig. 2B). The number of unique users submitting tickets increased from similar numbers by NGS and non-NGS users in 2011 (91 vs. 67, respectively) to 4 times the number of NGS users in 2016 (846 vs. 173, respectively).

Another major factor in explaining the difference in NGS and non-NGS ticket numbers is that users from NGS projects submitted nearly twice as many tickets per user as did users from non-NGS projects (Fig. 2C). Between 2011 and 2016, NGS users submitted 4.0 ± 0.11 tickets per user annually, while non-NGS users submitted 2.2 ± 0.07 tickets per user annually.

To better understand the nature of this nearly 2-fold difference, we randomly selected 100 NGS-related support tickets submitted each year between 2013 and 2016. Of the randomly selected tickets, roughly one-third were requests for maintenance or modifications of project-related computing and/or storage allocations, while 13% were requests for software tool installations or support (Supplementary Table S3).

### More installations and updates of NGS-related software

**Figure 3:**
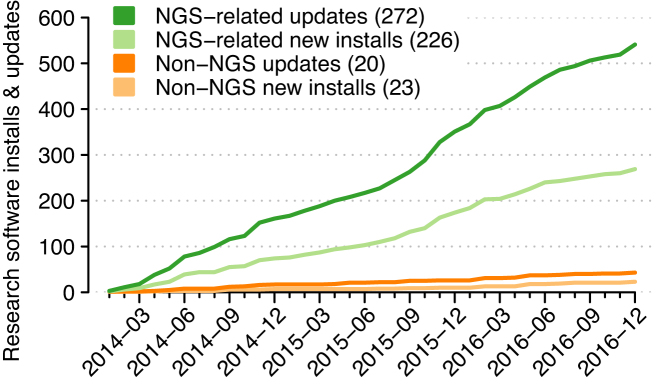
Research software installations and updates by application experts at UPPMAX between the start of 2014 and the end of 2016. Shown are cumulative numbers of updates and new installations for bioinformatics software and resources (green tones) and for other domain-specific software (orange tones), with categorical totals given in the legend. Scheduled updates of database resources are excluded, as are installations and updates of general-purpose software such as compilers, interpreters (including Perl, Python, R, and Matlab), and system tools.

Between 2014 and 2016, UPPMAX application experts performed 541 installations and updates of research-related software tools and resources such as databases (Fig. 3; see the Methods section for additional information on definitions and exclusions). More than 11 × as many NGS-related installations and updates were performed than installations and updates of tools and resources used by non-NGS projects (498 vs. 43, respectively). There was no difference in the relative number of installations vs. updates by research type (Fisher exact test, *P*= 0.34). NGS-related tools and resources were installed or updated at the rate of 13.8/month over this three-year period, while installations and updates of other tools and resources averaged 1.2/month over this same period. The pace of NGS-related installations and updates has accelerated from 12.0/month during 2014 to 14.8/month during 2015 and 2016.

### Monthly core hour usage now equivalent between NGS and non-NGS projects

**Figure 4:**
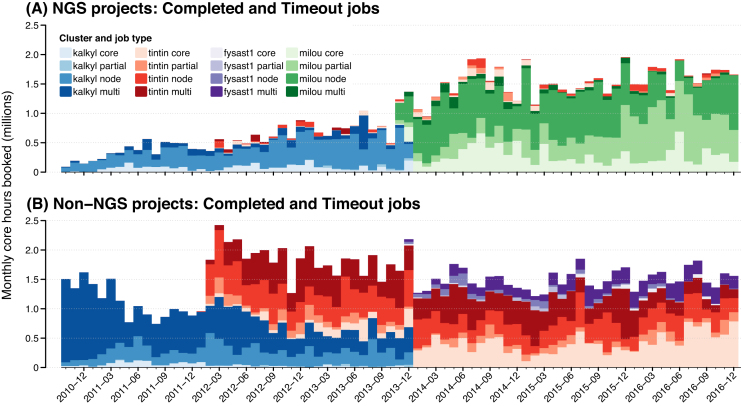
Monthly core hours booked by completed or timeout jobs run by projects at UPPMAX, by cluster and job type, from October 2010 through the end of 2016. A) NGS projects. B) Non-NGS projects. Job booking types: *core* - single core; *partial* - partial set of available cores on node; *node* - complete node, all cores; *multi* - more than 1 node. For cluster information, see Table 1; for job state fractions, see Supplementary Fig. S1.

The number of core hours (job duration × number of allocated central processing unit cores, Equation (1)) consumed monthly by SLURM jobs in both NGS and non-NGS projects approached and occasionally exceeded 2M (M = million) core hours/month for all jobs in each project type (Supplementary Fig. S1A, S1B). Considering jobs with completed or timeout end states (jobs that did not fail and were not cancelled), both projects regularly exceeded 1.5M core hours/month at the end of this study (full height of stacked bars in Fig.4A and 4B). Core hour usage by NGS projects grew from <200K/month toaround 2M/month, while over the same period, core hour usage by non-NGS projects was 1-1.5M/month by late 2010 and 1.5-2M/month at the end of 2016.

The trends in monthly core hour usage by projects parallel the trends in active project numbers presented above. There has been rapid growth in core hours used by NGS projects to the present level (Fig. 4A), contrasted with moderate growth by non-NGS projects from already high levels (Fig. 4B). Discrete changes apparent in monthly core hour trends occur because of the commissioning and decommissioning of computing clusters (Table 1).

From the last quarter of 2010 through the end of 2016, NGS projects submitted 6.6M jobs that consumed 80.6M core hours, while non-NGS projects submitted 9.2M jobs that consumed 121.2M core hours (Table 3). Mean job sizes in core hours were comparable between research project types, as were median job sizes (Table 3).

**Table 3:** SLURM job numbers and core hours at UPPMAX for NGS and non-NGS projects, from October 2010 through the end of 2016

Project type	Number of jobs	Total core hours	Mean core hours/job	Median core hours/job
NGS projects	6,626,228	80,622,505	12.2 ± 0.05	0.49 [0.11, 2.16]
Non-NGS projects	9,225,861	121,225,241	13.1 ± 0.09	0.56 [0.31, 0.85]

Excluded are very short jobs (<60 seconds) and failing jobs due to system error. See text for more details. Mean is ± s.e.m.; median includes the 25th and 75th quartiles.

### More core hours used by jobs that terminate with timeout in non-NGS projects

The fraction of core hours used by jobs that terminate with different end states also differs between project types. A SLURM job at UPPMAX has one of five end states: a *Completed* job terminated autonomously within the requested wall time limit; a job receives *Timeout* when terminated by SLURM for exceeding its requested wall time limit; a *Cancelled* job was terminated by the user; a *Failed* job suffered an autonomous error, perhaps because a software tool exited abnormally or available RAM was exceeded; and *Node Fail* indicates an internal error related to cluster hardware or software. *Node Fail* jobs (∼1% of core hours) are excluded from all plots; see the Methods section for more details.

Roughly one-quarter to one-third of monthly core hours in NGS projects were used by NGS jobs that terminated with Timeout, while roughly one-third to one-half of jobs in non-NGS projects end with Timeout (Supplementary Fig. S1A, S1B). We attribute this difference to the widespread availability of task-continuation support in software tools used by non-NGS projects, which is nearly completely lacking from NGS software tools, so that the large majority of core hours used by Timeout jobs in NGS projects are wasted. We discuss this further below.

### NGS jobs book fewer cores when more RAM per core is available

For this analysis, we divided jobs into four types based on the number of cores booked by the job: *core*, with a single core booked; *partial*, with >1 core but less than a complete node; *complete*, which booked a complete node; and *multi*, which booked multiple complete nodes. We also restricted our analysis to jobs with *Completed* and *Timeout* end states (see above).

Following the move of NGS projects from the (relatively) low-memory Kalkyl cluster (3 GiB/core, 24 GiB/node) to the high-memory Milou cluster (8 GiB/core, 128 GiB/node) at the end of 2013, NGS projects booked more core hours via jobs requesting less than a complete node (Fig. 4A). In 2013 on Kalkyl, 15.5% of NGS core hours were booked by core and partial jobs, in contrast to 2016 on Milou, when 65.4% of NGS core hours were booked by core and partial jobs. NGS jobs tend to require more RAM independent of the number of cores (see below), so most of this shift was due to the availability of increased RAM per core on Milou, which enabled more memory-demanding jobs to be run on fewer cores.

There was also an increase in core hours booked by single-core jobs over this time, in both NGS projects (Fig. 4A) and non-NGS projects (Fig. 4B). A portion of this increase in NGS projects reflects methodological innovations, such as computing-intensive statistical methods that use approximate Bayesian computation [e.g., 14]. Methodological innovations may also be responsible for some of a similar shift toward bookings with fewer cores in non-NGS research projects over this same period observed on the Tintin cluster (21.8% of core hours accumulated by core and partial jobs in 2013 on Tintin vs. 54.3% in 2016; Fig. 4B), which occurred without a corresponding increase in RAM per core.

### NGS projects rarely book multinode jobs

Multinode jobs were rarely used by NGS projects (3.5% of core hours in 2015, 1.6% in 2016; Fig. 4A) because few software tools used in NGS research are capable of multinode parallelism via, e.g., Open MPI [15]. In most cases, multinode jobs were booked by NGS projects as a result of user error. In contrast, MPI support is common in tools used by non-NGS projects and roughly one-third to one-half of core hours in non-NGS projects being accumulated by multinode jobs (45.6% in 2013, 43.9% in 2015, 31.3% in 2016; Fig. 4B).

### NGS jobs use more cores and more RAM

Here, we compare and contrast resource usage of jobs run by NGS and non-NGS projects in terms of both cores and RAM. Resource usage reflects actual usage of cores and memory as determined by direct monitoring of running jobs, rather than the amount of resources booked via job control examined in the previous sections. The resources booked for a job represent upper bounds on core and RAM resource usage over the life of a job, while actual resource usage is typically lower. See the Methods section for further details of data collection and Equations (2) and (3) for calculating resource usage.

**Figure 5:**
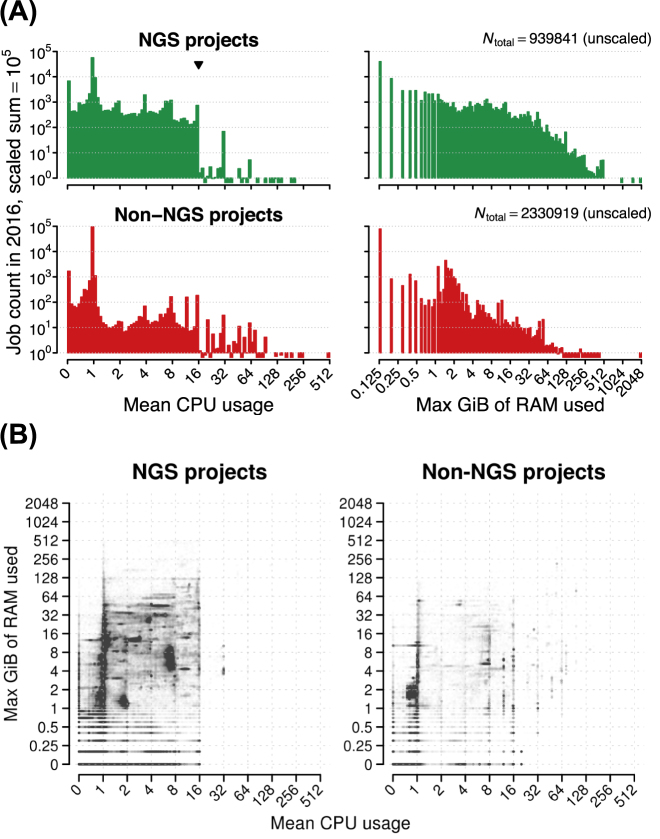
Resource usage by 3.27M jobs run by NGS and non-NGS projects at UPPMAX during the calendar year 2016. Resource usage was determined via monitoring. A) Histograms of core and RAM usage, with each range divided into 100 bins and bin counts scaled to sum to 10^5^ within each panel to facilitate comparisons (bin count × 10^5^/*N*_total_). Non-zero bins where the scaled count <1 show marks below the 10^0^ line. In the upper left panel, the inverted triangle marks the number of cores equivalent to a full node on the Milou cluster. B) Joint core and RAM usage, with each job plotted as a single dot with \raise.17ex∼1% opacity.

The majority of jobs in both NGS and non-NGS projects used a single core or less (70.5% and 97.5% of all jobs, respectively). For those jobs using up to a full node, a greater proportion of jobs used multiple cores in NGS projects than in non-NGS projects (Fig. 5A, left panels). The same was true for memory usage; a greater proportion of jobs used more RAM in NGS projects than in non-NGS projects (Fig. 5A, right panels).

These differences are all the more striking when we examine the joint distribution of job core and memory usage (Fig. 5B). NGS jobs use both more cores and more RAM than jobs in non-NGS projects, up to the limits provided by a single node.

### NGS projects run less efficient jobs, but active monitoring helps

We define efficiency of a single job as the fractional usage of booked resources, expressed as a percentage. We consider usage efficiency for cores (Equation (2)) and memory (Equation (3)) separately and also calculate an aggregate efficiency metric for each job (Equation (6)) that takes into account both core and memory efficiency. We calculate mean efficiencies per project, and we calculate the weekly per project type with Equations (7)–(9). See the Methods section for details on calculating resource usage efficiency.

**Figure 6:**
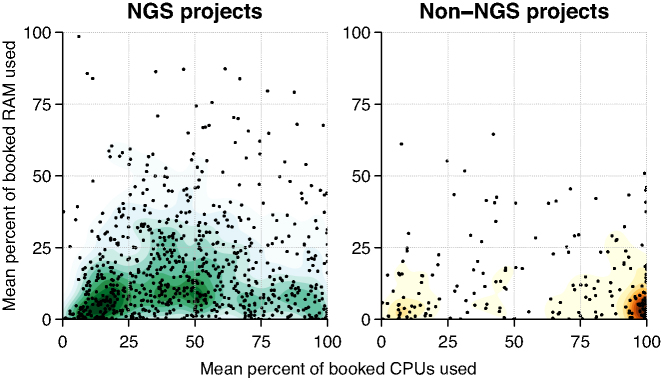
Efficiency of core and memory usage in UPPMAX projects by project type. Efficiency is measured as the average usage of requested cores and the maximum utilized amount of requested RAM; see the Methods section for further details. (Left) NGS projects’ mean percentage of booked core and memory resources for all jobs in a project during the calendar year 2016. (Right) Non-NGS projects’ mean percentage of booked core and memory resources for all jobs in a project during the same one-year period.

NGS projects had lower median core usage efficiency (44%, first and third quartiles [23%, 67%]) than non-NGS projects (73% [28%, 97%]) (Fig. 6). Conversely, median RAM usage efficiency is higher in NGS projects (12% [5%, 26%]) than in non-NGS projects (7% [2%, 18%]), reflecting greater memory usage by NGS jobs generally.

**Figure 7:**
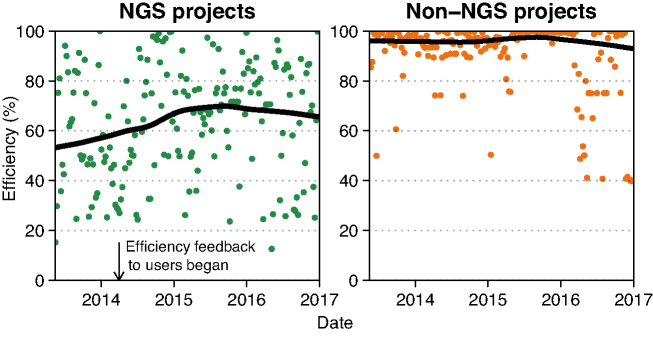
Weekly median efficiency of all jobs at UPPMAX by project type. Each dot is the median efficiency of all jobs run during that week, weighted by the fraction of core hours used per job overlapping that week; see the Methods section for details. The horizontal trend line is the smoothed LOESS regression of the dots. Also shown in the left plot is the time point where UPPMAX began providing feedback on job efficiency within projects whenever a user request for additional core hours was made.

When we began weekly monitoring of job efficiencies in 2013, the difference in job efficiencies between NGS and non-NGS projects was clear (Fig. 7). Further analysis revealed that for many NGS jobs, at least some inefficiencies were the result of easily corrected user error, through misunderstanding computational demands, misspecifying options to tools, or misbooking jobs through SLURM. During the first half of 2014, we began analyzing job efficiency whenever an NGS project PI applied for a continuation or increase in core hour allocation and would make the requested core hour allocation contingent on increasing job efficiency, if warranted. We also provided additional tools so that users could monitor their own resource usage (Supplementary Fig. S2A, S2B). We observed a steady increase in job efficiency for the first year after monitoring began, and efficiency stabilized in NGS projects at 70–75%. Active monitoring remains a central part of our monitoring strategy, and we continue to develop tools to aid users in improving job efficiency.

## Discussion

These comparisons reveal the following characteristics that differentiate HPC cluster usage for NGS research from that for non-NGS research at UPPMAX:

- Hosting NGS research carries a large administrative burden, with increased effort arising from management of core hour and storage allocations, user support tickets, and software installations.
- NGS jobs require more RAM than non-NGS jobs, regardless of job size, with single-core NGS jobs requiring more RAM/core and occasional jobs requiring 256 GiB shared memory/node or more.
- NGS jobs very rarely span multiple nodes and can make effective use of partial nodes down to single cores if there is sufficient RAM/core.
- NGS jobs use HPC resources less efficiently than non-NGS jobs; some efficiency can be gained with user education, but some progress is not possible due to lack of maturity in NGS software tools.

We discuss these in more detail below and conclude with recommendations for HPC clusters hosting NGS research computing projects.

### Effective hosting of NGS research carries a large administrative burden

Early in the development of academic NGS research in Sweden, UPPMAX and SciLifeLab determined to lower barriers to entry of new research groups by maintaining a great deal of flexibility in computing and storage allocations for research projects, as computing and storage demands have proven to be quite variable over NGS project lifetimes [e.g., 21]. As the number of active NGS projects and users has grown (Fig.1), this flexibility has resulted in a significant administrative burden on UPPMAX staff that has scaled roughly linearly with the number of projects. To offset this increase, UPPMAX staff have begun exploring some degree of automation for project and resource extensions and increases, as well as automating requests for medium-term high-performance scratch storage, with the goal of easing some more repetitive tasks while providing additional services.

Another result of easing entry to HPC computing for new research groups has been an influx of many new users having limited familiarity with command line interfaces and Linux. UPPMAX provides some access to its clusters via ThinLinc [16] but encourages users to become more familiar with Linux, the Linux command line, and shell scripting and runs courses to meet this need.

Even with such training, users have difficulties installing the tools, packages, and modules required for their research. Repositories associated with major scripting languages ease this somewhat (Python [17], Perl [18], R [19], and [20]. However, many NGS software tools are not available through such repositories and have installation procedures that can vary greatly from tool to tool and even between tool versions. This reflects the breadth of experience of NGS software tool developers but also presents considerable challenges to inexperienced users.

Largely driven by user requests, application experts at UPPMAX have expended considerable effort installing NGS software tools (Fig. 3) that are made available system wide via the Lmod module system [22]. The number of tools currently installed has made migration to new clusters labor intensive, so a comprehensive package system that simplifies the installation of tools made available via Lmod is desirable. EasyBuild [23] is one such system and is widely used to install non-NGS software on HPC clusters. The amount of bioinformatics software available via EasyBuild, at first very low, is continually increasing. UPPMAX staff will consider adopting EasyBuild in the coming months.

All of these factors jointly contribute to the number of support tickets submitted by NGS users (Fig. 2). A more hands-off approach would have resulted in less administrative effort, but it is unlikely that this would have fostered the amount of NGS research hosted at UPPMAX to date. The Linux and HPC expertise of NGS users is increasing, but we do not anticipate that the computation experience of a typical NGS user will approach that of a typical non-NGS user for several years to come.

### HPC clusters should be configured to run NGS jobs effectively

Given the high RAM demands of many NGS jobs together with the lack of distributed computation in NGS tools (Fig. 5), clusters with less than about 32 GiB of shared memory available to jobs will have difficulties hosting NGS projects. When larger amounts of RAM are available within single nodes, NGS jobs can make effective use of partial nodes (Fig. 4A), providing booking of partial nodes is supported by the job control system. High RAM demands by NGS jobs extend down to jobs run on single cores (Fig. 5A), where 4-6 GiB RAM/core are required for many such jobs, providing the job control system allows booking of single-core jobs.

Very few NGS jobs use multiple nodes compared to non-NGS jobs ( Figs. 4, 5), and many of the core hours booked as multinode jobs by NGS projects are the result of user inexperience. For NGS-dedicated systems, our experience shows that high-performance node-to-node interconnects such as InfiniBand are not often used. High-performance connections to high-performance storage, on the other hand, is of utmost importance.

Cloud computing represents one possible way to provide flexible computing architecture for NGS research, but it remains unclear whether cloud resources, especially those with sufficient RAM and storage, will be cost-effective for NGS analyses [24]. Our experience with local cloud computing for NGS research is limited. We provide support for virtualization via Singularity [25] for interested research groups.

### NGS users need guidance to use HPC resources efficiently

NGS projects at UPPMAX had lower resource usage efficiency (Fig. 6), largely the result of user inexperience. Particularly egregious examples of inefficiency can be readily addressed, e.g., booking a complete node but using a single core and little RAM (Supplementary Fig. S2A). However, for the many less straightforward cases, increasing efficiency requires greater familiarity with tool options, job control options, and knowledge of resource usage for similar jobs in the past.

The solution UPPMAX has adopted to address these inefficiencies shifts responsibility to the users and is three-fold: (1) monitor resource usage of all jobs and provide a tool (jobstats [26]; example plots in Supplementary Fig. S2A, S2B) that allows users to examine resource usage by running and completing jobs; (2) develop efficiency metrics (see the Methods section, Equations (1)–(11)) and apply these metrics to actively monitor the efficiency of resource usage by projects and users; and (3) make project allocation extensions contingent on efficiency, e.g., a request for a temporary increase in monthly core hours would not be granted or would be granted only, in part, if a project or user has consistently run jobs inefficiently. This approach has been effective; median efficiency of NGS jobs has increased since monitoring started in 2013 (Fig. 7).

However, there are other sources of inefficiency that are difficult for users to control. Heterogeneous resource usage by bioinformatics workflows is one such source. For example, a multithreaded step may be followed by an extended single-core step or steps may differ greatly in memory requirements. Additionally, jobs expected to consume large amounts of memory are likely to underuse memory, as swap space is very limited on computing nodes and jobs reaching 100% memory usage are at risk of failure. Tools that allow for specifying memory limits are relatively few in number and sorely needed. Finally, some memory inefficiency is unavoidable simply due to the granularity of resource availability on HPC clusters.

### NGS software tools could mature in a few specific ways

NGS software tools perform a wide range of computational tasks while managing very large quantities of data input and output [21]. New methodologies, new approaches, and novel research areas will continually give rise to new NGS software tools written by developers with widely varying levels of experience. This contrasts with non-NGS research software such as Gaussian [27], VASP [28], and others, which typically have large code bases, long lifetimes, and ample opportunities to mature. Aside from obvious general recommendations, such as code profiling, we suggest three ways in which development of NGS software tools could mature to further increase the efficiency of HPC cluster usage.

First, NGS software tools could provide the ability to continue tasks in progress. Roughly one-fourth of core hours used by NGS projects at UPPMAX, 400K–500K hours/month, are wasted because jobs terminated with Timeout (Supplementary Fig. S1A). The ability to continue common tasks such as read mapping, variant calling, and sequence alignments would help avoid this wastage. Non-NGS projects regularly have jobs terminate with Timeout (Supplementary Fig. S1B) because non-NGS research software tools such as GROMACS [29] and Amber [30] have supported task continuation for several years. A few NGS tools (e.g., those that use approximate Bayesian computation for statistical inference [31]) support task continuation on an *ad hoc* basis, but formal intermediate checkpoints are found in only a handful of multistage tools such as the genome assemblers MaSuRCA [32] and ABySS [33].

Second, NGS software tools could provide the ability to limit the amount of RAM used. NGS tools that allow the user to specify memory limits are few in number; Java-based tools and the assembler SPAdes [34] are notable exceptions. Even experienced users employ trial-and-error to discover these limits, leading to more wasted hours. At the least, guidance for expected memory usage should be provided in documentation wherever possible.

Third, NGS software tool developers could support installation frameworks such as EasyBuild [23] and Bioconda [35] when distributing tools in addition to providing access to source bundles or prebuilt binaries within repositories. Such frameworks have extensive support within end-user and HPC communities; given the very large number of NGS tools installed monthly (Fig. 3), this could save HPC center staff considerable effort.

## Recommendations

To conclude, we provide some recommendations for HPC clusters that host NGS research, based on our experience hosting both NGS and non-NGS computing projects at UPPMAX. For hardware and job control systems:

- Provide 6+ GiB/core and 100+ GiB shared memory/node.
- Job control systems should allow users to book partial nodes, down to single cores.
- Make high-performance, high-capacity storage accessible with high bandwidth.
- Multinode jobs are rare, so high-performance internode fabrics such as InfiniBand may not be required.

For user and application support:

- Provide courses to introduce both Linux and the cluster environment.
- Plan for software installations; NGS software tools have widely varying levels of developer sophistication.
- Monitor NGS project efficiency, and enable users to do so as well.
- Allow flexibility in core-hour and storage allocations.
- Make resource extensions contingent on efficiency, not core hours usage.
- Consider using installation frameworks such as EasyBuild.

For software tool developers and software distribution:

- Support task continuation.
- Allow for metering memory usage and document expected memory requirements.
- Support installation frameworks such as EasyBuild and Bioconda when distributing tools.

The NGS research community is large, dynamic, and continues to grow rapidly. HPC centers can do much with their expertise to support these new users and foster exciting research.

## Methods

### Active projects, project storage, and principal investigators

We retrieved project start and end dates, PIs, and storage usage from internal databases. We did not begin explicit daily logging of project storage until 2013.

The identity of project PIs has been standardized in stages, first by assigning a local identity number and later by linking the identity number with a countrywide authentication service. However, for older projects, PI names were entered manually and could have multiple alternate spellings. To determine PI identity for older projects, we constructed a Levenshtein distance matrix (R function adist [36]) for all unmatched names against all PIs with an identity number. We assigned close matches (edit distance ≤7) to existing PIs after manual confirmation. We manually linked the remaining unmatched PI names to existing PIs where possible and created new PI identities where necessary.

### Inferring project types for support tickets

User-submitted support tickets are not explicitly assigned to local usernames or projects at UPPMAX, so project type was inferred for each support ticket. We matched the submitter email address to an UPPMAX username with user databases. We excluded tickets for which the submitter email address could not be matched, tickets that requested user accounts, and tickets submitted by UPPMAX system experts. We assigned each username to a predominant project type (NGS or non-NGS) based on project membership; very few users were active in both types of projects.

### Software installations

Software packages installed by application experts at UPPMAX are made available to users via the Lmod module system [22]. We used changelog entries to calculate numbers of new installations and updates of NGS and non-NGS research-related software and databases. We defined an *installation* as the installation of a tool or resource not previously available on UPPMAX clusters and an *update* as the installation of an updated version of a tool or resource already available on UPPMAX clusters. A partial collection of installation procedures is available at [37].

Examples of NGS-related tools installed at UPPMAX include BWA [38], ABySS [33], Salmon [39], GATK [40], and Kraken [41]. Examples of tools for other types of research installed at UPPMAX include GROMACS [29], Gaussian [27], Amber [30], VASP [28], and RSPt [44]. We excluded general-purpose tools such as compilers, interpreters (e.g., Perl, Python, R, Matlab), editors, and general-purpose libraries such as libcurl [45]. We also excluded database updates scheduled via crontab.

### Compute resource usage and efficiency

Details of computing jobs run via the SLURM job management system [12] were collected from internal databases. The complete jobs dataset, from October 2010 through December 2016, contained 23.6M jobs that booked 240M core hours. Several types of jobs are excluded from the data presented in the body of this article. Jobs with less than 60 seconds of wall time were excluded; these represented 18.1% of total jobs but just 0.024% of total core hours. We also excluded jobs run by course projects for student instruction (37.2K jobs, 544K core hours) and jobs run by sequencing core facility projects at SciLifeLab (764K jobs, 12.6M core hours). Finally, we excluded 23.1K jobs (0.1%) that consumed 2.85M core hours (1.2%) that terminated with the SLURM condition *Node Fail*, indicating a system error beyond user control. The data underlying Fig. 4 and Supplementary Fig. S1 are available as Supplemental Data D1.

For each job, we calculated core and memory usage as the total usage of resources booked over the duration of a job. A given job *j* books κ_*j*_ cores and μ_*j*_ GiB RAM, with the number of booked cores specified explicitly and the amount of booked memory dependent upon other user requests, such as the GiB/core available on the specific cluster and node type on which the job is run (Table 1). The number of core hours *H*_*j*_ booked by a job that ran for *h*_*j*_ hours of wall time is

(1) \begin{eqnarray*}
H_{j}&= h_{j}\kappa _{j}
\end{eqnarray*}

For each SLURM job at UPPMAX, the current core usage (fraction busy for each core, 0−100%) and memory usage (RAM in GiB) are logged every 5 minutes. For job *j* with τ_*j*_ ≥ 1 log entries, core usage at time point *t* is *c*_*j*_(*t*), the sum of the fraction busy of all booked cores at *t*, and memory usage is *m*_*j*_(*t*) with a maximum of μ_*j*_. Core and memory usage for the entire job are calculated as:

(2) \begin{eqnarray*}
C_{j} = \frac{1}{\tau _{j}} \sum _{t=1}^{\tau _{j}}c_{j}(t)\quad 0 \le C_{j}\le \kappa _{j}
\end{eqnarray*}

(3) \begin{eqnarray*}
M_{j}= \max _{t \in \tau _{j}}\ m_{j}(t)\quad 0 \le M_{j}\le \mu _{j}
\end{eqnarray*}

We calculated memory usage as the maximum used at any time point because the amount of available memory μ_*j*_ serves as a hard upper bound for the memory available to complete a job successfully.

Core usage efficiency of a job Ξ_*j*_ is the core usage divided by the number of booked cores:

(4) \begin{eqnarray*}
\Xi _j = \frac{C_{j}}{\kappa _{j}} \quad 0 \le \Xi _j\le 1
\end{eqnarray*}

Memory usage efficiency is the memory usage divided by the amount of memory booked:

(5) \begin{eqnarray*}\Gamma _j = \frac{M_{j}}{\mu _{j}} \quad 0 \le \Gamma _j\le 1
\end{eqnarray*}

Job efficiency is the maximum of either efficiency measure:

(6) \begin{eqnarray*}E_j = \max \left( \Xi _j, \Gamma _j\right) \quad 0 \le E_j\le 1
\end{eqnarray*}

Mean efficiencies for a set of jobs *J*_*S*_ weight each job by job length and, for core and job efficiency, also weight each job by booked cores:

(7) \begin{eqnarray*}\Xi _S = \frac{\sum _{j} C_{j}\times \tau _{j}\kappa _{j}}{\sum _{j} \tau _{j}\kappa _{j}} \quad j \in J_S
\end{eqnarray*}

(8) \begin{eqnarray*}\Gamma _S = \frac{\sum _{j} M_{j}\times \tau _{j}}{\sum _{j} \tau _{j}} \quad j \in J_S
\end{eqnarray*}

(9) \begin{eqnarray*}E_S = \frac{\sum _{j} E_j\times \tau _{j}\kappa _{j}}{\sum _{j} \tau _{j}\kappa _{j}} \quad j \in J_S
\end{eqnarray*}

For summaries of resource efficiencies by project type, averages for each individual project were calculated using Equations (7)–(9), with *J*_*S*_ containing the jobs run by each project during a specified time period. Project averages were then used to estimate a kernel distribution [46,47]. The contribution of each project *P* to the kernel was given a weight ω_*P*_ determined by the number of core hours *H*_*P*_ used by the project during the time period, with the smallest active projects given a minimum weight:

(10) \begin{equation*}
\omega _P= \left\lbrace \begin{array}{@{}l@{\quad }l@{}}1 & 0 < H_P\le 10 \\\log _{10}H_P& \text{otherwise} \end{array}\right.
\end{equation*}

To examine trends in job efficiency over time, we calculated the weighted median weekly job efficiency. To calculate the weekly contribution for each job, we calculated the efficiency of each job *E*_*j*_ using Equation (6) and calculated the weight ω_*j*_ for each job using the following procedure. The set of jobs that run during a given week *W* is designated *J*_*W*_. For each job *j* ∈ *J*_*W*_, the fraction of its total wall time that overlaps week *W* is *o*_*j*_. A single job can have *o*_*j*_ > 0 for multiple consecutive weeks with the constraint that for each job ∑_*W*_*o*_*j*_ = 1. The weight for job *j* is the amount of its core hours accumulated during week *W*:

(11) \begin{eqnarray*}\omega _{j}&= o_jH_{j}
\end{eqnarray*}

Median weighted efficiency for each week is calculated using these weights and efficiency of each job *E*_*j*_ calculated using Equation (6).

## Availability of supporting data

Scripts for analysis and producing figures are available at [48] as are some smaller datasets. Anonymized versions of the storage, job, and efficiency databases and a snapshot of the above-mention scripts are available at [49] from the *GigaScienceGiga*DB server. In the datasets we are releasing, we include anonymized records for 25M jobs, with efficiency metrics for 15M of those jobs, run by 2,124 unique projects, as well as storage usage data from 1,384 days covering 2,848 unique projects. The jobstats tool is available at [26].

## Abbreviations

HPC, high-performance computing; NGS, next-generation sequencing; PI, principal investigator; RAM, random access memory; SNIC, Swedish National Infrastructure for Computing; T UPPMAX, Uppsala Multidisciplinary Center for Advanced Computational Science.

## Competing interests

The authors declare that they have no competing interests.

## Supplementary Material

GIGA-D-17-00352_Original_Submission.pdf

GIGA-D-17-00352_Revision_1.pdf

GIGA-D-17-00352_Revision_2.pdf

Response_to_Reviewer_Comments_Original_Submission.pdf

Response_to_Reviewer_Comments_Revision_1.pdf

Reviewer_1_Report_(Original_Submission) -- Stephen Newhouse 19 Jan 2018 Reviewed

Reviewer_2_Report_(Original_Submission) -- Krithika Bhuvaneshwar 29 Jan 2018 Reviewed

Reviewer_3_Report_(Original_Submission) -- Jay Lofstead 05 Feb 2018 Reviewed

Supplemental material

## References

[1] Koboldt DC, Steinberg KM, Larson DE The next-generation sequencing revolution and its impact on genomics. Cell. 2013;155(1):27–38. 24074859 10.1016/j.cell.2013.09.006 PMC3969849

[2] Bleidorn C Third generation sequencing: technology and its potential impact on evolutionary biodiversity research. Systematics and Biodiversity. 2016;14(1):1–8.

[3] Mignardi M, Nilsson M Fourth-generation sequencing in the cell and the clinic. Genome Medicine. 2014;6(4):31. 25031621 10.1186/gm548 PMC4062057

[4] Roberts RJ, Carneiro MO, Schatz MC The advantages of SMRT sequencing. Genome Biology. 2013;14(7):1–4. 10.1186/gb-2013-14-7-405 PMC3953343 23822731

[5] Ekblom R, Wolf JBW A field guide to whole-genome sequencing, assembly and annotation. Evolutionary Applications. 2014;7(9):1026–42. 25553065 10.1111/eva.12178 PMC4231593

[6] Eid J, Fehr A, Gray J Real-Time DNA sequencing from single polymerase molecules. Science. 2009;323(5910):133–38. 19023044 10.1126/science.1162986

[7] Kuleshov V, Xie D, Chen R Whole-genome haplotyping using long reads and statistical methods. Nature Biotechnology. 2014;32(3):261. 10.1038/nbt.2833 PMC4073643 24561555

[8] Pabinger S, Dander A, Fischer M, A survey of tools for variant analysis of next-generation genome sequencing data. Briefings in Bioinformatics. 2014;15(2):256–278. 23341494 10.1093/bib/bbs086 PMC3956068

[9] Harrison R, Li Y, Măndoiu I Bioinformatics Research and Applications: 11th International Symposium, ISBRA 2015 Norfolk, USA, June 7-10, 2015 Proceedings,vol. 9096 Springer; 2015.

[10] Post DE, Votta LG Computational science demands a new paradigm. Physics Today. 2005;58(1):35–41.

[11] Spjuth O, Bongcam-Rudloff E, Dahlberg J, Recommendations on e-infrastructures for next-generation sequencing. GigaScience. 2016;5(1):1–9. 10.1186/s13742-016-0132-7 PMC4897895 27267963

[12] Yoo AB, Jette MA, Mark G SLURM: Simple Linux Utility for Resource Management. In: Lecture Notes in Computer Science;2003p. 44–60.

[13] Lampa S, Samuel L, Martin D Lessons learned from implementing a national infrastructure in Sweden for storage and analysis of next-generation sequencing data. GigaScience. 2013;2(1):9. 23800020 10.1186/2047-217X-2-9 PMC3704847

[14] Wegmann D, Leuenberger C, Neuenschwander S, ABCtoolbox: a versatile toolkit for approximate Bayesian computations. BMC Bioinformatics. 2010;11(1):116. 20202215 10.1186/1471-2105-11-116 PMC2848233

[15] Gabriel E, Fagg GE, Bosilca G, Open MPI: goals, concept, and design of a next generation MPI implementation. In: Proceedings, 11th European PVM/MPI Users’ Group Meeting Budapest, Hungary; 2004 p. 97–104.

[16] Cendio ThinLinc https://www.cendio.com/thinlinc. Accessed 1st Feb 2018

[17] The Python Package Indexhttps://pypi.python.org. Accessed 1st Feb 2018

[18] The Comprehensive Perl Archive Network https://www.cpan.org/. Accessed 1st Feb 2018

[19] CRAN Archive https://cran.r-project.org/. Accessed 1st Feb 2018

[20] Bioconductor https://www.bioconductor.org/. Accessed 1st Feb 2018

[21] Muir P, Li S, Lou S, The real cost of sequencing: scaling computation to keep pace with data generation. Genome Biol. 2016;17(1):53. 27009100 10.1186/s13059-016-0917-0 PMC4806511

[22] Geimer M, Markus G, Kenneth H, Modern scientific software management using easybuild and lmod. In: 2014 First International Workshop on HPC User Support Tools; 2014.

[23] Hoste K, Timmerman J, Georges A, EasyBuild: building software with ease. In: Proceedings of the 2012 SC Companion: High Performance Computing, Networking Storage and Analysis SCC ’12, Washington, DC, USA: IEEE Computer Society; 2012; p. 572–582..http://dx.doi.org/10.1109/SC.Companion.2012.81.

[24] Emeras J, Varrette S, Plugaru V Amazon Elastic Compute Cloud (EC2) vs. in-house HPC platform: a cost analysis. IEEE Transactions on Cloud Computing. 2016;.

[25] Kurtzer GM, Sochat V, Bauer MW Singularity: scientific containers for mobility of compute. PLoS ONE. 2017; 12(5):1–20. 10.1371/journal.pone.0177459 PMC5426675 28494014

[26] UPPMAX jobstats https://github.com/UPPMAX/jobstats. Accessed 1st Feb 2018

[27] Guassian http://gaussian.com/. Accessed 1st Feb 2018

[28] Vienna Ab initio Simulation Package https://www.vasp.at/. Accessed 1st Feb 2018

[29] Abraham MJ, Murtola T, Schulz R GROMACS: high performance molecular simulations through multi-level parallelism from laptops to supercomputers. SoftwareX. 2015;1–2:19–25.

[30] Case DA, Cheatham TE, Darden T, The Amber biomolecular simulation programs. Journal of Computational Chemistry. 2005;26(16):1668–88. 16200636 10.1002/jcc.20290 PMC1989667

[31] Csilléry K, Blum MGB, Gaggiotti OE, Approximate Bayesian computation (ABC) in practice. Trends in Ecology & Evolution. 2010;25(7):410–18. 20488578 10.1016/j.tree.2010.04.001

[32] Zimin AV, Marçais G, Puiu D The MaSuRCA genome assembler. Bioinformatics. 2013;29(21):2669–77. 23990416 10.1093/bioinformatics/btt476 PMC3799473

[33] Simpson JT, Wong K, Jackman SD ABySS: a parallel assembler for short read sequence data. Genome Res. 2009;19(6):1117–23. 19251739 10.1101/gr.089532.108 PMC2694472

[34] Bankevich A, Nurk S, Antipov D, SPAdes: a new genome assembly algorithm and its applications to single-cell sequencing. Journal of Computational Biology. 2012;19(5):455–77. 22506599 10.1089/cmb.2012.0021 PMC3342519

[35] Grüning B, Dale R, Sjödin A Bioconda: a sustainable and comprehensive software distribution for the life sciences. bioRxiv. 2017;https://www.biorxiv.org/content/early/2017/10/27/207092. 10.1038/s41592-018-0046-7 PMC11070151 29967506

[36] R Core Team. R: A Language and Environment for Statistical Computing. R Foundation for Statistical Computing, Vienna, Austria; 2017; https://www.R-project.org/.

[37] UPPMAX install methods https://github.com/UPPMAX/install-methods. Accessed 1st Feb 2018

[38] Li H, Durbin R Fast and accurate long-read alignment with Burrows-Wheeler transform. Bioinformatics. 2010;26(5):589. 20080505 10.1093/bioinformatics/btp698 PMC2828108

[39] Patro R, Duggal G, Love MI, Salmon provides fast and bias-aware quantification of transcript expression. Nat Meth. 2017; 14(4):417–19. 10.1038/nmeth.4197 PMC5600148 28263959

[40] McKenna A, Hanna M, Banks E The Genome Analysis Toolkit: a MapReduce framework for analyzing next-generation DNA sequencing data. Genome Res. 2010; 20(9):1297–1303. 20644199 10.1101/gr.107524.110 PMC2928508

[41] Wood DE, Salzberg SL Kraken: ultrafast metagenomic sequence classification using exact alignments. Genome Biology. 2014;15(3):R46. 24580807 10.1186/gb-2014-15-3-r46 PMC4053813

[44] Wills JM, Alouani M, Andersson P Full-potential electronic structure method. Energy and Force Calculations with Density Functional and Dynamical Mean Field Theory. Berlin: Springer; 2010.

[45] https://curl.haxx.se/libcurl/. Accessed 1st Feb 2018

[46] Wand MP Fast computation of multivariate kernel estimators. J Comput Graph Stat. 1994;3(4):433.

[47] Bäcklin CL, Andersson C, Gustafsson MG Self-tuning density estimation based on Bayesian averaging of adaptive kernel density estimations yields state-of-the-art performance. Pattern Recognition. 2018;78:133–43.

[48] https://github.com/douglasgscofield/pubs/tree/master/Dahlo-et-al-1. Accessed 1st Feb 2018

[49] Dahlö M, Scofield DG, Schaal W, Supporting data for “Tracking the NGS revolution: managing life science research on shared high-performance computing clusters.”. GigaScience Database. 2018;https://doi.org/10.5524/100421. 10.1093/gigascience/giy028 PMC5928410 29659792

