# Supplementary material for: Tracking the NGS revolution: managing life science research on shared high-performance computing clusters
Source: Gigascience. 2018 Apr 5;7(5):giy028. doi: 10.1093/gigascience/giy028 (PMC5928410; doi:10.1093/gigascience/giy028)

GigaScience, 2017, 1–3

doi: xx.xxxx/xxxx
Manuscript in Preparation
Research

RESEARCH

Supplemental Information for "Tracking the NGS revolution: managing life science research on shared high-performance computing clusters"

Martin Dahlö[1,2,3,*,‡], Douglas G. Scofield[2,4,†,‡], Wesley Schaal[1,2,3] and Ola Spjuth[1,2,3]

[1]Science for Life Laboratory, Uppsala University, SE-750 03 Uppsala, Sweden and [2]Uppsala Multidisciplinary Center for Advanced Computational Science, Uppsala University, SE-751 05 Uppsala, Sweden and [3]Department of Pharmaceutical Biosciences, Uppsala University, SE-751 24 Uppsala, Sweden and [4]Department of Ecology and Genetics: Evolutionary Biology, Uppsala University, SE-752 36 Uppsala, Sweden

[*]martin.dahlo@scilifelab.uu.se
[†]douglas.scofield@ebc.uu.se
[‡]Contributed equally.

Compiled on: December 21, 2017.
Draft manuscript prepared by the author.

Table S1. Additional UPPMAX compute cluster infrastructure

HPC Cluster	Nodes	Total cores	RAM/node	Usage
Bianca (2015–2018)	100	1600	128 GiB	For sensitive NGS data, primarily human whole genome sequencing
Irma (2015–2018)	250	4000	256 GiB	New NGI sequencing platform NGS production system
Halvan (2011–2015)	1	64	2 TiB	High-memory jobs, primarily genome assembly from NGS data

Table S2. Additional UPPMAX storage infrastructure

HPC Storage	Capacity	Format	Description
Castor (2016–2019)	2 PiB	Ceph	Storage for sensitive NGS data
Lupus (2016–2019)	1 PiB	Lustre	Storage for sequencing platform NGS data

Table S3. Topics of support tickets submitted by NGS users, 2013–2016. 100 support tickets submitted by NGS users were randomly selected from all support tickets in each year. Support ticket topics are: *Allocations*, user requests for extensions or increases in compute or storage allocations; *Applications*, software tool support or installation requests; *Password*, password reset requests; and *Other*, other topics. The relative increase in allocations requests during 2016 was due to a temporary policy change which decreased project expiration times, since reversed.

Year		Support ticket topic		
	Allocations	Applications	Password	Other
2013	32	14	3	51
2014	30	14	12	44
2015	32	12	7	49
2016	44	12	5	29

Figure S1. Monthly core hours booked by projects at UPPMAX, by job end state, from October 2010 through the end of 2016. (A) NGS projects. (B) Non-NGS projects. The marked increase in early 2012 for non-NGS projects is due to the installation of the Tintin cluster (see Figure 4 in the main text). Job end states: *Completed*, terminated normally within the user-requested time limit; *Timeout*, cancelled by job control after exceeding the user-requested time limit; *Cancelled*, manually terminated by the user while running; *Failed*, terminated abnormally. See the main text for further details.

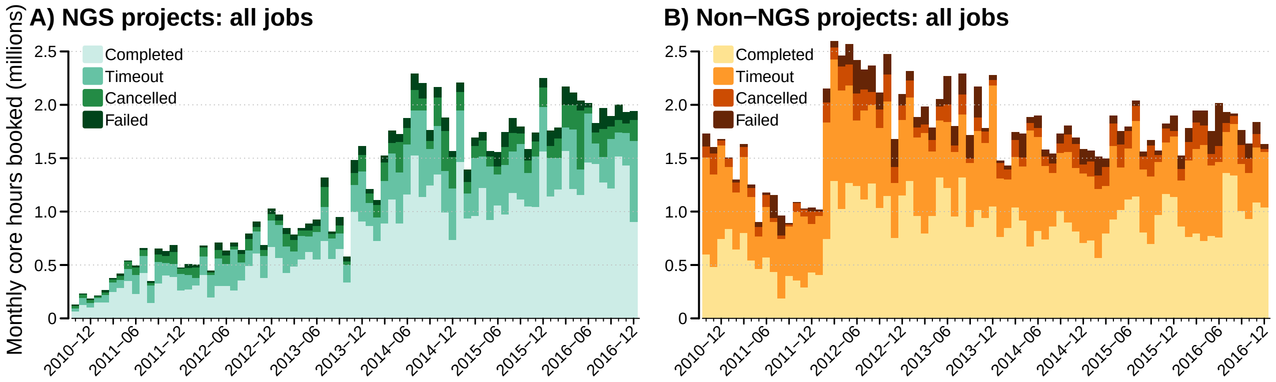

Figure S2. Job resource usage efficiency examples. These jobstats (https://github.com/UPPMAX/jobstats) plots have traces for RAM usage (in black, scale is left y-axis) and core usage (in blue, scale is right y-axis). The extents of the left and right axes represent the amount of resources booked for the job, while the extent of the x-axis represents the total run time of the job. (A) A job with low efficiency. A full node with 16 cores has been booked, but only a single core is used throughout the job and very little RAM is used. This job should have been booked on a single core. (B) A job with high efficiency. A whole node has been booked and all cores are used throughout the job. Memory usage is low, but overall job efficiency is determined by core *or* memory efficiency. An example of a job with high memory efficiency would be one that uses > 100 GiB RAM at some time point within the job. See the main text for further details on calculating resource usage and efficiency, and the jobstats repository for more on jobstats.

(A)

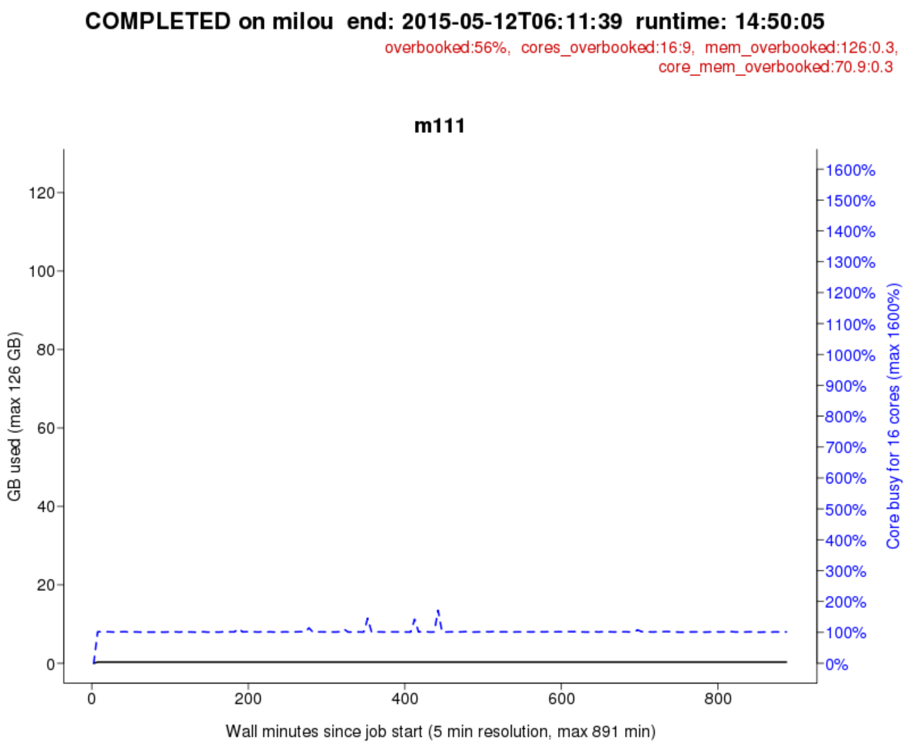

(B)

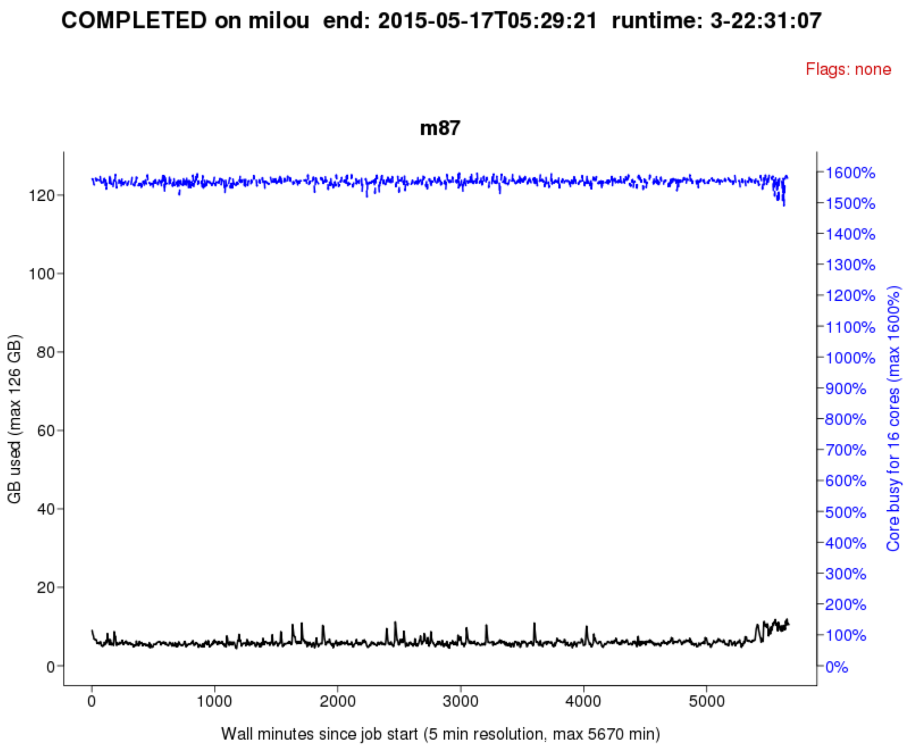

Supplement: Supplemental material [file giy028_supp.pdf]
