# Supplementary material for: Tracking the NGS revolution: managing life science research on shared high-performance computing clusters
Source: Gigascience. 2018 Apr 5;7(5):giy028. doi: 10.1093/gigascience/giy028 (PMC5928410; doi:10.1093/gigascience/giy028)
Supplement: Response_to_Reviewer_Comments_Original_Submission.pdf [file giy028_response_to_reviewer_comments_original_submission.pdf]

Author's Response To Reviewer Comments

Subject: Your submission to GigaScience - GIGA-D-17-00352
GIGA-D-17-00352
Tracking the NGS revolution: managing life science research on shared high-performance computing clusters
Martin Dahlö; Douglas Glen Scofield; Wesley Schaal; Ola Spjuth
GigaScience

Dear Mr Dahlö,

Your manuscript "Tracking the NGS revolution: managing life science research on shared high-performance computing clusters" (GIGA-D-17-00352) has been assessed by our reviewers. Based on these reports, and my own assessment as Editor, I am pleased to inform you that it is potentially acceptable for publication in GigaScience, once you have carried out some essential revisions suggested by our reviewers.

Their reports, together with any other comments, are below. Please also take a moment to check our website at https://giga.editorialmanager.com/ for any additional comments that were saved as attachments.

Once you have made the necessary corrections, please submit a revised manuscript online at:

https://giga.editorialmanager.com/

If you have forgotten your username or password please use the "Send Login Details" link to get your login information. For security reasons, your password will be reset.

Please include a point-by-point within the 'Response to Reviewers' box in the submission system. Please ensure you describe additional experiments that were carried out and include a detailed rebuttal of any criticisms or requested revisions that you disagreed with. Please also ensure that your revised manuscript conforms to the journal style, which can be found in the Instructions for Authors on the journal homepage.

The due date for submitting the revised version of your article is 06 May 2018.

We look forward to receiving your revised manuscript soon.

Best wishes,

Scott Edmunds, Ph.D.
GigaScience
www.gigasciencejournal.com

Reviewer reports:
Reviewer #1: This is a great resource and point of reference for everyone involved in NGS

processing, HPC management - or thinking of setting up a cluster to manage NGS workloads. The community would benefit from more reports like this from other groups around the world - need more like this showing realities of NGS and demands on HPC.

>>> Thank you for your comments!

Reviewer #2: In recent years, scientists have been analyzing every part of the genome. Next generation sequencing (NGS) technology based research is a relatively young and fast growing/changing area of research that deals with big data. As more and more data is generated, data storage, handing and analyses becomes increasingly challenging. The authors have gathered and analyzed data from the HPC clusters at UPPMAX computing centre at Uppsala University, Sweden. They have compared and contrasted the growth, administrative burden and cluster usage of NGS projects with projects from other sciences.

The authors have touched upon a very important topic, and I commend the authors for doing this analysis. My comments are below:

>>> Thank you for your helpful comments, we address these below.

- The data has been very well tracked and documented, and results have been presented well. All of the points mentioned by the authors related to task allocation, algorithm development, make complete sense. In this regard the recommendations provided by the authors are very helpful.

- In addition to cluster usage and optimization, there are other important factors that go hand in hand with NGS data handing - data storage and cost aspect. The authors mention in the abstract that storage used by NGS projects has grown rapidly since 2013. The also have tracked information related to data storage. NGS data storage is a challenging problem because the input data is large, the intermediate data is very large, and the output data is often small. I think it deserves more than just one line "High-performance, high-capacity storage accessible with high bandwidth". Does the UPPMAx center store all NGS data forever, or do they recommend purging all the data every few months ? Are there other recommendations the authors can provide from this experience ?

>>> We have added an additional paragraph at the end of the "Rapid growth in NGS projects, research groups and storage" subsection to provide more details of the solutions that we have implemented.

- Another aspect is the cost. Is the cost of storage and use of HPC clusters overlaid to the users ? For small /medium academic center that may not full dedicated funding for their HPC clusters, are there recommendations to estimate the cost of an NGS project, and how to optimize cost savings?

>>> Because of the way infrastructure is funded in Sweden, users of UPPMAX and other SNIC centres do not pay fees, and we have added a sentence in the introduction clarifying this. Based on experience with securing contracts for storage, storage costs can vary widely and providing backup for a fraction of storage, which we consider a necessity for NGS projects, can inflate

costs considerably. High-performance storage matching our requirements can easily cost more than the compute nodes, and we now make this point in the manuscript. The many issues surrounding the provisioning of NGS storage infrastructure is deserving of a separate study.

- The authors themselves mention that for NGS pipelines, one tool maybe multi-threaded while the next tool may need to run on a single core. Is it worth implementing a framework that does automatic scaling or automatic allocation of nodes/cores for NGS analysis based on the tool requirements? I believe there are a number of such frameworks available. Would that make the administrative burden lighter ?

>>> We provide some of these frameworks via our module system and research groups can use them if they wish. User inexperience probably prevents more widespread adoption of such frameworks, but we leave it to each research group to consider the costs and benefits for themselves. As is our policy with other aspects of NGS methodology, we remain agnostic as to which methods groups chose to use to improve efficiency.

- Title says "Tracking the NGS revolution". This cannot be complete without discussing cloud computing. It will be helpful to discuss whether the UPPMAX computing center is planning to test NGS analysis using cloud based infrastructure in the future, what their future plans and expectations are, etc.

>>> UPPMAX and other SNIC centres have begun providing cloud computing resources to groups that which to explore this, but our experience with NGS projects on cloud is thus far limited. In particular, our cloud infrastructure for both RAM and disk storage in insufficient for most NGS projects. We provide local virtualisation via Singularity for groups that wish to use it, but there as well our direct experience is limited. We have provided an additional (short) paragraph in the Discussion including these points.

Reviewer #3: This paper presents a review of the usage of Swedish research computers for both NGS (Next-Generation Sequencing) and non-NGS workloads in an effort to better understand how the shifting workloads and user base are affecting both the administrative burdens and how new machines should be configured.

The paper presents a nice, detailed set of observations supported by statistical evidence provided in the paper about job sizes, core hours used, and other metrics for both types of workloads. The conclusions drawn are reasonable and driven from the evidence provided. These conclusions also are largely in line with general thought on the topic, but provide hard data points based on experiences rather than anecdotal evidence.

The recommendations in the paper on how to better serve the different user communities is good, but potentially a little troubling. For example, the non-NGS workloads really benefit from high speed networking, such as InfiniBand. Moving to Ethernet is a significant performance penalty. While this is a proportionally shrinking part of the overall workload, it can suffer. The US DOE is being told to make the post-exascale machines capable of handling both kinds of workloads effectively. This paper provides some insights into how we may need to configure these machines and some of the tradeoffs we will have to deal with. These insights are valuable to both

DOE and others having to do this system design.

>>> Moving to Ethernet is certainly a performance hit, and we hope it is clear from the text that we have based this recommendation on our observations of NGS projects and that it is meant as a point for consideration.

This is the right kind of report at the right time.

>>> We agree! Thank you for the very kind comments.
