# Supplementary material for: Tracking the NGS revolution: managing life science research on shared high-performance computing clusters
Source: Gigascience. 2018 Apr 5;7(5):giy028. doi: 10.1093/gigascience/giy028 (PMC5928410; doi:10.1093/gigascience/giy028)
Supplement: Response_to_Reviewer_Comments_Revision_1.pdf [file giy028_response_to_reviewer_comments_revision_1.pdf]

Author's Response To Reviewer Comments

Hello, and thank you for your comments! We have addressed them and corrected to the best of our abilities. See in-line response to comments below:

"Please add a Supporting Data Section just after the software section at the end of the paper, mentioning that test data and code snapshots are available from the GigaScience GigaDB server. Then you then need to cite this as reference 14 as follows:

Dahlö, M; Scofield, D; Schaal, W; Spjuth, O (2018): Supporting data for "Tracking the NGS revolution: managing life science research on shared high-performance computing clusters" GigaScience Database. http://dx.doi.org/10.5524/100421"

*** Done, added the reference and adjusted the text in our supporting data section.

"In addition, please register any new software application in the SciCrunch.org database to receive a RRID (Research Resource Identification Initiative ID) number, and include this in your manuscript software section. This will facilitate tracking, reproducibility and re-use of your tool. And include any other RRIDs (ftp://penguin.genomics.cn/pub/10.5524/RRID/RRIDlist.pdf) and author ORCID details in the paper."

*** We are not presenting software for others to use as a tool, it is being provided for openness. The snapshot in the GigaScience DB should fill that need. We have added ORCID entries for all authors right after the author list on the first page of the paper.
