# Supplementary material for: Tracking the NGS revolution: managing life science research on shared high-performance computing clusters
Source: Gigascience. 2018 Apr 5;7(5):giy028. doi: 10.1093/gigascience/giy028 (PMC5928410; doi:10.1093/gigascience/giy028)
Supplement: Reviewer_3_Report_(Original_Submission) -- Jay Lofstead — 05 Feb 2018 Reviewed [file giy028_reviewer_3_report_(original_submission).pdf]

Reviewer Report

Title: Tracking the NGS revolution: managing life science research on shared high-performance computing clusters

Version: Original Submission **Date:** 05 Feb 2018

Reviewer name: Jay Lofstead

Reviewer Comments to Author:

This paper presents a review of the usage of Sweedish research computers for both NGS (Next-Generation Sequencing) and non-NGS workloads in an effort to better understand how the shifting workloads and user base are affecting both the administrative burdens and how new machines should be configured.

The paper presents a nice, detailed set of observations supported by statistical evidence provided in the paper about job sizes, core hours used, and other metrics for both types of workloads. The conclusions drawn are reasonable and driven from the evidence provided. These conclusions also are largely in line with general thought on the topic, but provide hard data points based on experiences rather than anecdotal evidence.

The recommendations in the paper on how to better serve the different user communities is good, but potentially a little troubling. For example, the non-NGS workloads really benefit from high speed networking, such as InfiniBand. Moving to Ethernet is a significant performance penalty. While this is a proportionally shrinking part of the overall workload, it can suffer. The US DOE is being told to make the post-exascale machines capable of handling both kinds of workloads effectively. This paper provides some insights into how we may need to configure these machines and some of the tradeoffs we will have to deal with. These insights are valuable to both DOE and others having to do this system design.

This is the right kind of report at the right time.

Methods

Are the methods appropriate to the aims of the study, are they well described, and are necessary controls included? Yes

Conclusions

Are the conclusions adequately supported by the data shown? Yes

Reporting Standards

Does the manuscript adhere to the journal's guidelines on minimum standards of reporting? Yes

Statistics

Are you able to assess all statistics in the manuscript, including the appropriateness of statistical tests used? Yes, and I have assessed the statistics in my report.

Quality of Written English

Please indicate the quality of language in the manuscript: Acceptable

Declaration of Competing Interests

Please complete a declaration of competing interests, considering the following questions:

- Have you in the past five years received reimbursements, fees, funding, or salary from an organisation that may in any way gain or lose financially from the publication of this manuscript, either now or in the future?
- Do you hold any stocks or shares in an organisation that may in any way gain or lose financially from the publication of this manuscript, either now or in the future?
- Do you hold or are you currently applying for any patents relating to the content of the manuscript?
- Have you received reimbursements, fees, funding, or salary from an organization that holds or has applied for patents relating to the content of the manuscript?
- Do you have any other financial competing interests?
- Do you have any non-financial competing interests in relation to this paper?

If you can answer no to all of the above, write 'I declare that I have no competing interests' below. If your reply is yes to any, please give details below.

I declare that I have no competing interests

I agree to the open peer review policy of the journal. I understand that my name will be included on my report to the authors and, if the manuscript is accepted for publication, my named report including any attachments I upload will be posted on the website along with the authors' responses. I agree for my report to be made available under an Open Access Creative Commons CC-BY license (http://creativecommons.org/licenses/by/4.0/). I understand that any comments which I do not wish to be included in my named report can be included as confidential comments to the editors, which will not be published.

I agree to the open peer review policy of the journal

To further support our reviewers, we have joined with Publons, where you can gain additional credit to further highlight your hard work (see: https://publons.com/journal/530/gigascience). On publication of

this paper, your review will be automatically added to Publons, you can then choose whether or not to claim your Publons credit. I understand this statement.

Yes
